# Challenges facing a Rheumatology journal: Role and credentials of editorial board members

**DOI:** 10.31138/mjr.28.2.99

**Published:** 2017-06-27

**Authors:** Vinod Ravindran, Durga Prasanna Misra, Vir Singh Negi

**Affiliations:** 1 Centre for Rheumatology, Calicut, Kerala, India,; 2 Department of Clinical Immunology, Sanjay Gandhi Postgraduate Institute of Medical Sciences (SGPGIMS), Lucknow, India,; 3 Department of Clinical Immunology, Jawaharlal Institute of Postgraduate Medical Education & Research (JIPMER), Puducherry, India

**Keywords:** Rheumatology, journal, publishing, editorial board

Managing a scientific Journal is a challenging task, more so for clinician scientists as they are tasked with the onerous responsibility of guiding an established means of scientific communication. We would like to share our experiences of editing for the *Indian Journal of Rheumatology* (www.indianjrheumatol.com).

The *Journal* is an official publication of the Indian Rheumatology Association which represents clinicians practicing in this field. The *Journal* has been in existence for over two decades with 4 quarterly issues and two supplements (one thematic supplement with guest editors and the other containing conference abstracts) every year. In brief, the main challenges faced by the *Journal* are: attracting good quality original articles, getting indexed in Medline, maintaining the standards of the *Journal* both online and print, promoting the culture of scientific writing and publications and keeping the younger generation of rheumatologists from India interested in the editorial work for the *Journal* .

For the IJR, the responsibility of selecting the core group of editors (currently four Associate Editors and sixteen Assistant Editors) as well as National and International Editorial Board (EB) members lies with the Editor-in-Chief (EiC). Selecting the members of an EB is crucial to the future of a journal. The members of the IJR EB too, in common with many other journals, function without any extra remuneration in addition to their day job. People in the current EB of the *Journal* have several features in common: they are dynamic and creative, are willing to present the Journal and its credentials elsewhere as well as show initiative of soliciting articles for the Journal. Few of them have been involved with the *Journal* for a long time either as reviewers or authors. The EiC has a tenure of three years; however, the continuity of key decisions regarding the *Journal* is maintained by having the immediate past editor as the emeritus editor who gives input in all EB meetings. The current core EB of the *Journal* has members from all regions in India and includes rheumatologists based in academic institutes as well as in private practice. One member each is specifically responsible for pediatric rheumatology, bio-statistics and technical aspects related to submitted manuscripts.

The current national EB of the *Journal* consists of people who are seniors in this field and they bring together their years of experience in operational aspects and also acts as the overall quality control. The current international EB of the *Journal* has a global representation from members who are leading experts in their respective fields.

**Figure 1:**
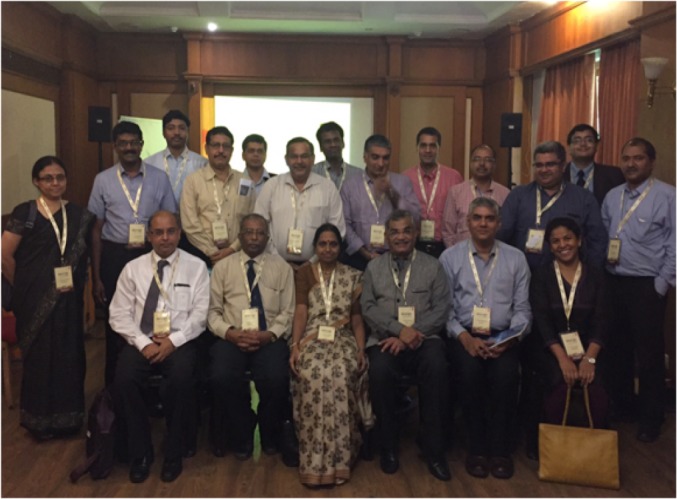
The Editorial Board of the Indian Journal of Rheumatology at the annual meeting of the Indian Rheumatology Association at Kochi, India, in November 2016.

The EB on the whole is responsible for adhering to the strictest ethical aspects of editing and publishing in line with the latest recommendations from Committee on Publication Ethics (COPE) and other such reputed organizations such as the International Committee of Medical Journal Editors (ICMJE) and the Council of Science Editors (CSE). ^1^ Members of the EB contribute towards a Journal by themselves supplying quality articles (both original and review articles) as well as guiding supplement issues of the journal. They aim to utilise their expertise in the subject as well as in the areas of ethical editing and publishing to enhance the quality of the Journal. The EB meets at regular intervals to chalk out the future of the Journal. In today’s world, with the distances of physical travel being bridged by the Internet, it is not unusual to have meetings using modalities such as video conferencing. Physical EB meetings are restricted to one per year, centered on the society’s annual national conference ( **Figure 1** ). These meetings are critical to setting out short-term goals and long-term agendas for the *Journal* . Short-term goals could revolve around a schedule for review articles for the next year or so. Long-term agendas would often include discussing the major aforementioned challenges to the *Journal.* Another point of discussion is how to improve turnaround times, which is often a matter of concern for authors. In general, prompt turnaround times are appreciated by authors, irrespective of the ultimate outcome of their submission. Improving the visibility and profile of the *Jounal* is another concern of the EB. The members of the EB may improve visibility of their journal by citing quality articles from the Journal in their own papers, so as to make readers all over the world aware that such a Journal exists and is producing work of this quality. However, they do this keeping in mind the ethical requirement to cite only highly relevant articles and avoid artificially boosting citations. Also, whenever they present at meetings, they may actively display their affiliation with the Journals as EB members so as to increase the awareness about the Journal and endorsing the quality of the same. In addition, EB members critically appraise the IJR’s website and also are a faculty for the workshops on scientific writing and publication which are conducted 2–3 times in a year.

To summarize, the EB plays a crucial role in guiding the future of a Journal such as IJR. Working as a team, not only the *Journal* has made major improvements in several areas, but has also been able to groom future generations of editors.
